# A machine learning approach for classifying date fruit varieties at the Rutab stage

**DOI:** 10.3389/fpls.2025.1678757

**Published:** 2025-11-26

**Authors:** Meshal Alfarhood, Nawaf Alsahw, Mohammed Almajed, Meshaal Alzahrani, Ahmad Alawfi, Meshal Alanazi, Abdalrahman Alalwan

**Affiliations:** Department of Computer Science, College of Computer and Information Sciences, King Saud University, Riyadh, Saudi Arabia

**Keywords:** dates, fruit classification, machine learning, YOLO, Rutab stage, image recognition, agricultural technology, and mobile application

## Abstract

**Introduction:**

Dates have long been a vital part of the cultural and nutritional heritage of arid regions, particularly in the Middle East. Among their ripening stages, the Rutab stage—an intermediate phase between the Khalal (immature) and Tamar (fully ripe) stages—holds unique significance in terms of taste, texture, and market value. However, the classification of Rutab varieties remains underrepresented in the literature.

**Methods:**

To address this gap, we present a pipeline that leverages machine learning to classify Rutab dates from images. A custom dataset comprising 1,659 images across eight popular Rutab types was collected, and several deep learning models were evaluated.

**Results and discussion:**

Among the tested models, YOLOv12 achieved the highest recall of 93%. The proposed system is deployed within a mobile application, aiming to promote cultural preservation and increase global awareness of the diversity found within date varieties.

## 1 Introduction

Dates are an integral part of agricultural and dietary systems in many regions, particularly in the Middle East and North Africa. With over 200 varieties, dates thrive in harsh environmental conditions such as extreme heat and water scarcity. These fruits have historically been crucial for the survival and development of ancient civilizations in desert regions worldwide (Jain and Johnson, 2015). Today, dates are widely recognized as a highly nutritious health food, rich in fructose—a natural sugar—as well as essential vitamins and dietary fiber. Additionally, dates are valued for their medicinal properties, including cholesterol-lowering effects and their role in preventing diseases such as cancer, diabetes, and cardiovascular conditions (Al-Dashti et al., 2021).

With a wide range of varieties, dates differ in size, shape, color, texture, and taste. The classification of these cultivars is crucial for quality control, market pricing, pest management, and consumer satisfaction (Rojas Santelices et al., 2025). Moreover, many in our current and future generations are unaware of the different types of dates and their seasons. Traditionally, dates classification has relied on manual inspection by experts, a process that is labor-intensive, time-consuming, and prone to human error. As the demand for automation in the agricultural sector grows, there is an urgent need for robust and efficient systems capable of classifying dates accurately and consistently.

Recently, machine learning (ML) has emerged as a transformative tool across many academic and industrial fields, providing powerful tools for pattern recognition, prediction, and classification. The agricultural sector, in particular, has experienced rapid growth in the application of ML techniques to address complex challenges, ranging from crop yield prediction to disease detection and quality grading of produce (Liakos et al., 2018). For fruit classification, ML algorithms have demonstrated considerable success in providing objective, rapid, and non-destructive evaluation. Various approaches, including Support Vector Machines (SVM), k-Nearest Neighbors (KNN), and increasingly, deep learning models like model based-Convolutional Neural Networks (CNNs), have shown high effectiveness in categorizing fruits based on their external and internal characteristics (Sahidullah et al., 2023). By learning from large datasets and subtle cues, these models suit dates classification and can improve efficiency and standardization.

**Figure 1:**
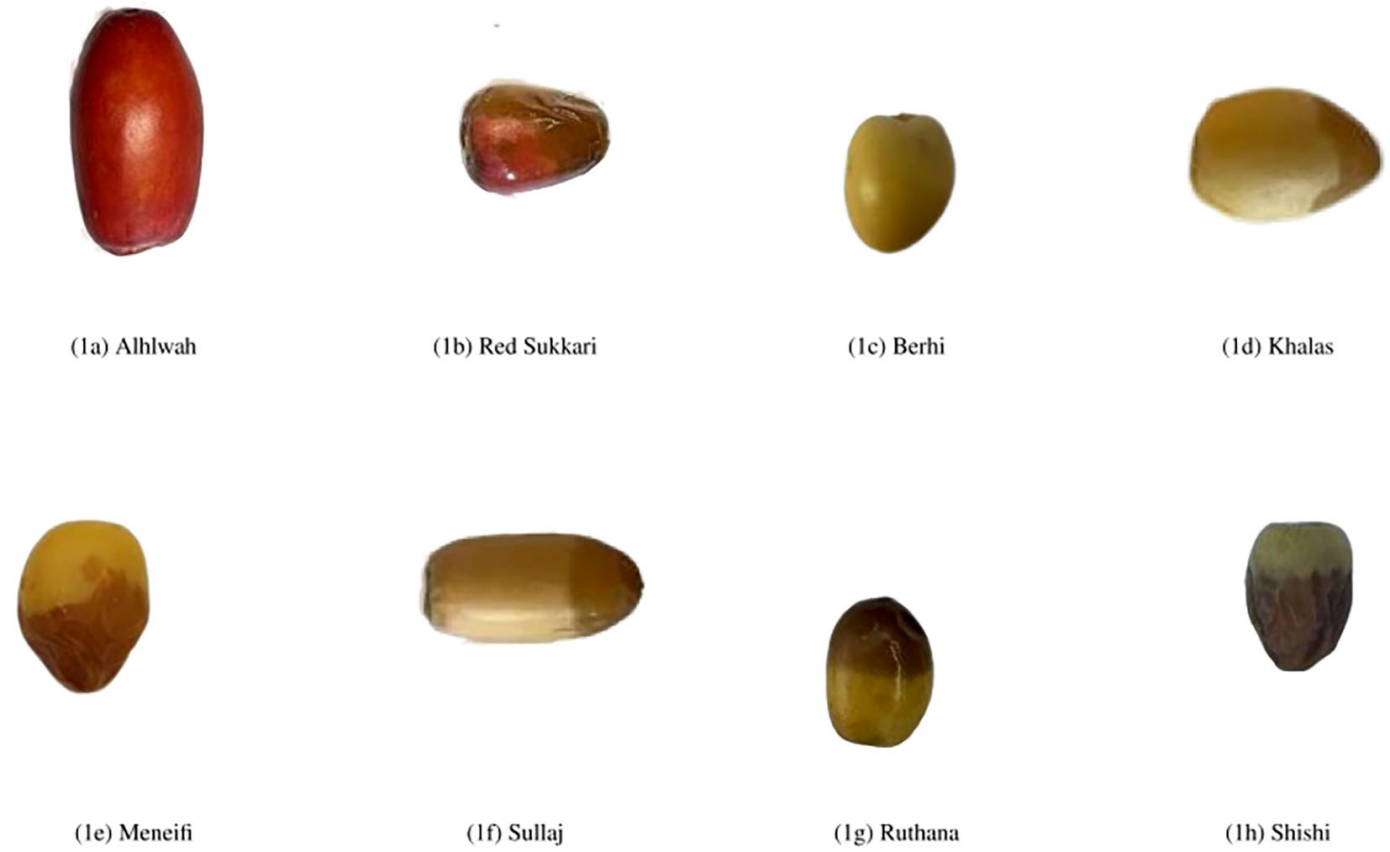
Representative sample images of the eight selected date varieties at the Rutab stage of ripening. The visual differences in color, texture, and shape across varieties highlight the classification challenge addressed in this study.

Most of the existing research in the domain of date fruit classification focuses on the final stage of dates and neglects the “Rutab” stage, which is the middle stage of date development between the Tamar (final) and Khalal (immature) stages (Altaheri et al., 2019). This gap is significant because many Rutab varieties share highly similar visual characteristics (such as color, shape, and texture), making them much harder to distinguish. In contrast, studies targeting other ripeness stages or more visually distinct classes often face a less challenging classification problem. In this paper, we aim to address this gap by using advanced machine learning techniques to classify “Rutab” dates. To achieve this, we created our own dataset specifically for “Rutab” dates, focusing on eight widely consumed varieties: Khalas, Sullaj, Alhlwah, Red Sukkari, Barhi, Ruthanah, Meneifi, and Shishi. **Figure 1** illustrates representative samples from each variety. In total, the dataset includes 1659 labeled images, ensuring a balanced representation across all eight Rutab types for training and evaluation purposes.

In addition, we fine-tuned five state-of-the-art deep learning models for this task: a baseline Convolutional Neural Network (CNN) (LeCun et al., 1998), ResNet-50 (He et al., 2016), EfficientNet (Tan and Le, 2019), YOLOv8 (Varghese and Sambath, 2024), and YOLOv12 (Tian et al., 2025). Among these, YOLOv12 demonstrated superior performance, achieving the highest recall of 93%, indicating its strong ability to correctly identify Rutab varieties across diverse conditions. To bridge research and practical application, the finalized model was deployed within a user-friendly mobile application, enabling real-time classification of Rutab dates in real-world settings.

To summarize, this paper’s primary contributions can be outlined in three main points:

- First, we created a unique dataset specifically focused on the “Rutab” stage, covering eight popular types of dates. Since there is limited work in the literature addressing this stage, our dataset serves as a valuable resource for future studies on date classification.
- Second, we explored and fine-tuned multiple deep learning techniques, proposing an effective pipeline for “Rutab” classification. Our approach achieved a high recall value of 93%, demonstrating its accuracy and reliability.
- Third, we integrated our findings into a mobile application, making it easy and accessible for users interested in classifying “Rutab” dates in real-world scenarios.

The remainder of this paper is organized as follows. Section 2 reviews related literature, highlighting significant studies and methodologies pertinent to our research. Section 3 details our methodology, covering the entire process from data collection to the deployment of the mobile application. In Section 4, we present and analyze our results, discussing key findings along with their limitations. Finally, Section 5 concludes the paper by summarizing the main outcomes and suggesting directions for future work.

## 2 Related work

The classification of date fruit has been a prominent area of research, with numerous studies employing diverse techniques for various applications, including automated harvesting systems and varietal identification. These efforts have also contributed valuable date fruit datasets to the research community. Beyond date fruits, the broader field of plant classification has seen significant advancements. Studies focusing on other plants, such as cherries and palm trees, have explored sophisticated techniques to enhance accuracy, often by merging multiple models or expanding limited datasets through Generative Adversarial Networks (GANs) for data augmentation. This rich body of work provides a strong foundation for our investigation into date fruit classification.

### 2.1 Approaches to date fruit classification

Koklu et al (Koklu et al., 2021). classified date fruits into genetic varieties using image analysis. Their research employed various machine learning methods, developing models with logistic regression (LR) and artificial neural network (ANN) techniques. The dataset for this study comprised 898 images across seven distinct date fruit types: Barhee, Deglet Nour, Sukkary, Rotab Mozafati, Ruthana, Safawi, and Sagai. The individual methods yielded accuracies of 91% for LR and 92.2% for ANN. Notably, combining these models into a stacking ensemble increased the accuracy to a successful 92.8%.

Meanwhile, Raed Sababa and Samy Abu-Naser (Sababa and Abu-Naser, 2024) utilized a pre-trained Convolutional Neural Network (ConvNeXtTiny). They trained their system on a dataset of 1,350 images to recognize nine date types. The deep learning model they developed achieved an outstanding accuracy of 99.44% on the test set. This high precision highlighted the model’s effectiveness in classifying diverse date varieties, which varied in size, shape, and sugar content, promising significant advancements in date fruit production and consumption.

Albarrak et al (Albarrak et al., 2022). also introduced a deep learning-based approach for classifying date fruits, aiming to develop an automated system using machine learning techniques. Their study incorporated methods such as image augmentation, decaying learning rates, model checkpointing, and hybrid weight adjustments to enhance accuracy. They compiled a dataset comprising eight date fruit varieties (Safawi, Khudri, Shishi, Ambir, Raziz, Mabroom, Khalas, and Lubana) to train the model. The proposed system, built on the MobileNetV2 architecture, achieved an impressive accuracy of 99%.

These classification models possess considerable real-world applicability. For instance, Faisal et al (Faisal et al., 2020). developed a harvesting system to estimate date fruit type and maturity level using computer vision (CV) and deep learning (DL). Their approach incorporated four different deep learning architectures: ResNet, VGG-19, Inception-V3, and NASNet. They utilized an online dataset of 8079 high-resolution images, covering five distinct date types and their maturity levels. The system demonstrated strong performance, with the ResNet model proving to be the most effective. Meanwhile, Adnan and Nasharuddin (2024) introduced an innovative Android-based mobile application designed to facilitate rapid identification of date fruit varieties and enhance user knowledge. By employing a transfer learning technique with a pre-trained neural network, their app successfully categorized nine date types, such as Ajwa, Medjool, and Rutab, achieving a remarkable 94.2% accuracy rate.

Morover, Almutairi et al (Almutairi et al., 2024). utilized the famous algorithm You Only Look Once (YOLO). They trained various models like YOLOv5, YOLOv7, and YOLOv8 on a dataset of 1735 images, representing nine distinct types of dates. They achieved remarkable results using YOLOv8, with a mean recall of 0.99%, precision of 0.991%, and a mean average precision (mAP) of 0.994%. On the other hand, Öznur Özaltın (2024) suggested image-feature-based machine learning algorithms to classify date fruit. The study used decision tree, K-nearest neighbors, artificial neural networks, and support vector machine algorithms, with different hyperparameters employed to classify date fruit. The results showed that the best algorithm was a 25-layer neural network, achieving a 93.85% test accuracy.

Additionally, Rybacki et al (Rybacki et al., 2024). proposed DateNET, an automatic classification model for different varieties of date palm fruits using a convolutional neural network (CNN). Their model focused on two key factors: geometric parameters and color differences of the dates. The dataset they used contained 500 images, with 100 images for each variety. Color-based classification achieved 85.24%, geometric classification 87.62%, and an impressive 93.41% when both factors were combined.

While these studies demonstrate impressive accuracy in classifying date varieties, it is important to note that most of them focus on the Tamar stage, where visual differences between varieties are more pronounced. Tamar dates typically exhibit distinct color, texture, and dryness, which provide clearer cues for image-based models. In contrast, Rutab dates, which are semi-ripe, often share similar color tones and smooth textures, making them harder to distinguish. This visual similarity among Rutab varieties introduces greater intra-class ambiguity and inter-class overlap, which can reduce classification performance even with advanced models. Our study in this paper addresses this gap by focusing specifically on the Rutab stage, where classification remains underexplored and more complex.

### 2.2 Classification techniques in other fruit and plant species

Other studies that are not directly related to dates can provide general insights into new algorithms and mechanisms. For example, Momeny et al (Momeny et al., 2020). presented a CNN-based hybrid pooling approach that can accurately classify cherry fruit into two classes. The research used an innovative CNN architecture that combines multiple pooling techniques, including average and max pooling. The model was evaluated using the CIFAR-10 dataset. As a result, the CNN model achieved a high classification accuracy rate of 99.4%. Also, Phan et al (Phan et al., 2023). proposed a system to classify tomatoes into three classes: mature, unripe, and damaged by utilizing four different deep learning models: YOLOv5, ResNet50, ResNet-101, and EfficientNet-B0. With 4,500 images in the dataset, YOLOv5 with ResNet-101 achieved the best performance among other combinations.

Moreover, Gulzar Yonis (Gulzar, 2023) developed a fruit image classification model using transfer learning with MobileNet-v2. The dataset comprised 26,149 images across 40 fruit classes. Through experimentation, their TL-MobileNetV2 model achieved 99% accuracy, effectively demonstrating its capability for robust fruit classification. Moreover, Safran et al (Safran et al., 2024). introduced DPXception, a lightweight CNN model designed for classifying date palm trees. They utilized a dataset of 2358 images representing four different date palm species. When tested against seven well-known CNN models, including ResNet50 and DenseNet201, DPXception achieved the highest accuracy at 92.9%.

Additionally, Zhang et al (Zhang et al., 2021). developed a deep learning-based model for classifying tree species using RGB optical images captured by an unmanned aerial vehicle (UAV). The approach combined advanced deep learning techniques with aerial imagery to identify individual tree species. The study employed CNN architectures such as AlexNet, VGG-16, and ResNet-50, using a dataset that included tree canopy images with both simple and complex backgrounds. The performance of these models was compared against traditional methods like K-nearest neighbor (KNN) and backpropagation (BP) neural networks. The analysis demonstrated that ResNet-50 was particularly effective for urban tree species classification based on RGB imagery.

**Table 1** provides a summary of the reviewed literature, detailing the domain, the number of images and classes in the datasets used, and the applied model that achieved the highest accuracy in each study.

**Table 1:** Comparative summary of related work, highlighting key aspects including the application domain, dataset size, and the classification models employed.

Work	Domain	Dataset	Adopted model
(Koklu et al., 2021)	Date Classification	898 images (7 classes)	Logistic Regression and Artificial Neural Network
(Sababa and Abu-Naser, 2024)	Date Classification	1350 images (9 classes)	ConvNeXtTiny
(Albarrak et al., 2022)	Date Classification	1717 images (8 classes)	MobileNetV2
(Faisal et al., 2020)	Date Classification	8079 images (5 classes)	ResNet
(Adnan and Nasharuddin, 2024)	Date Classification	1658 (9 classes)	Pre-trained TensorFlow Lite
(Almutairi et al., 2024)	Date Classification	1735 images (9 classes)	YOLOv8
(Özaltın, 2024)	Date Classification	898 images (7 classes)	Artificial Neural Network
(Rybacki et al., 2024)	Date Classification	500 images (5 classes)	DateNET (CNN)
(Momeny et al., 2020)	Cherry Classification	719 images (2 classes)	Convolutional Neural Network
(Phan et al., 2023)	Tomato Classification	4500 images (3 classes)	YOLOv5 and ResNet
(Gulzar, 2023)	Fruit Classification	26,149 images (40 classes)	MobileNetV2
(Safran et al., 2024)	Palm Tree Classification	2358 images (4 classes)	DPXception (CNN-based)
(Zhang et al., 2021)	Tree Classification	19,302 images (10 classes)	ResNet-50

This comparison provides context for the current study and underscores the novelty of focusing specifically on Rutab date classification using a custom-curated dataset and state-of-the-art deep learning techniques.

## 3 Materials and methods

**Figure 2:**
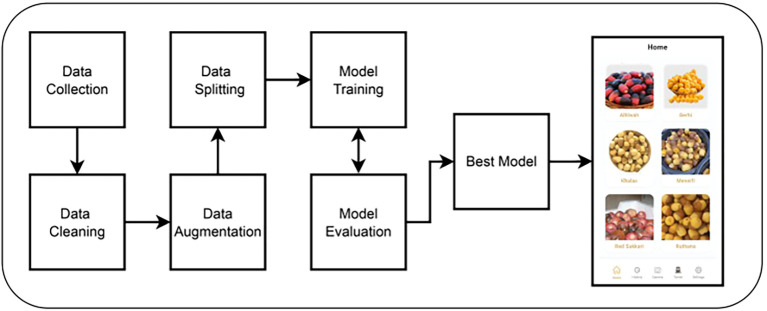
An overview of the proposed methodological pipeline, illustrating the seven key stages from data acquisition to deployment.

Our approach to classifying Rutab date varieties involves a multi-stage pipeline, as illustrated in **Figure 2**. The process begins with the construction of our own custom dataset, which we collected manually under controlled conditions. Following data acquisition, we applied a series of preprocessing steps. These included cropping to isolate the date fruit and resizing the images to a standardized input dimension suitable for deep learning models. To enhance model generalization, we employed various data augmentation techniques such as horizontal flipping and random rotation. The dataset was then partitioned into training, validation, and testing subsets using a stratified split to preserve class distribution across sets. Subsequently, we fine-tuned several state-of-the-art deep learning architectures, including CNN, YOLOv8, ResNet50, EfficientNetB0, and YOLOv12. The best-performing model—YOLOv12 in our case—was selected for integration into a mobile application designed for public use, enabling users to classify Rutab dates in real time. The following sections provide detailed descriptions of each step in our methodology, including dataset collection, data cleaning, data augmentation, data splitting, model training, model evaluation, and mobile development.

### 3.1 Data collection

To develop a robust and reliable classification system for Rutab dates, we first constructed our own custom dataset, as publicly available datasets focusing specifically on this intermediate ripening stage are currently lacking. The absence of such datasets presents a gap in the literature and limits the development of machine learning models tailored to this crucial phase of date fruit maturity.

Our dataset comprises a total of 1659 high-resolution images. These images represent eight widely recognized Rutab date varieties that are commonly cultivated and consumed in the Middle East. The selected varieties include Khalas, Sullaj, Alhlwah, Red Sukkari, Barhi, Ruthanah, Meneifi, and Shishi—all of which are culturally and commercially significant in the region.

To ensure the accuracy and reliability of class labels, each image was reviewed and verified by individuals with expert knowledge in dates classification, including local farmers and agricultural specialists familiar with the characteristics of Rutab varieties. **Table 2** presents the distribution of images across the eight Rutab types within our dataset.

**Table 2:** Distribution of the 1659 images in our collected dataset across the eight Rutab date varieties.

Rutab date type	Number of images
Alhalwah	109
Barhi	253
Khalas	229
Meneifi	208
Red Sukkari	133
Ruthana	271
Shishi	248
Sullaj	208
Total	1659

To ensure consistency and quality in image collection, Rutab date images were captured using a 12-megapixel smartphone camera in JPEG format at a resolution of 4032 × 3024 pixels. The photos were taken in a closed indoor room under bright lighting conditions provided by overhead fluorescent lights. The camera was positioned approximately 30 cm above the samples, and a plain white background was used to minimize visual distractions and enhance contrast.

### 3.2 Data cleaning

To ensure consistency in input dimensions and image quality, all collected images underwent a systematic preprocessing pipeline prior to model training, resulting in a final collected dataset of 1659 high-quality images. The preprocessing stage consisted of three main steps: image filtering, cropping, and resizing, as outlined below:

- Image Filtering: We performed a manual inspection of the initially collected dataset to remove images that did not meet quality standards. This included excluding images that were blurry, underexposed, overexposed, or exhibited significant occlusions. Additionally, images with distorted color profiles, irregular shapes, or excessively uniform backgrounds were excluded to improve the diversity and representativeness of the dataset. This filtering process was essential for ensuring that the deep learning models trained on this dataset could generalize well to real-world scenarios.
- Image Cropping: To maintain a consistent aspect ratio and eliminate unnecessary background variations, each image was cropped to a square format centered around the date fruit. This step helped focus the model’s attention on the relevant features of the Rutab dates.
- Image Resizing: Following cropping, all images were resized to a fixed resolution of 224 × 224 pixels. This dimension was selected to comply with the input size requirements of standard convolutional neural network architectures such as ResNet and EfficientNet, while also optimizing computational efficiency.

### 3.3 Data augmentation

To improve the model’s generalization to varied real-world scenarios, a consistent set of geometric data augmentation techniques was applied throughout the training process. This also helped reduce overfitting caused by the dataset’s limited size. The specific augmentation settings included the following:

- Rotation: Random rotation within a range of ±20° to account for angular variation during image acquisition.
- Translation: Random shift of up to ±10% along both horizontal and vertical axes to simulate positional variance.
- Scaling: Uniform scaling by up to ±10% to introduce scale invariance.
- Shearing: Geometric shearing up to ±5° to reflect distortions that may occur during handheld photography.
- Horizontal Flip: Applied with a probability of 50% to augment viewpoint diversity.

Color-based augmentations such as changes in hue, saturation, brightness, as well as composite techniques like mixup and mosaic, were deliberately excluded. This was based on the need to preserve the subtle chromatic and textural cues that are essential for distinguishing between Rutab types, many of which differ primarily in surface coloration and translucency. As such, the augmentation strategy prioritized geometric transformations that generalize spatial features while maintaining the color fidelity necessary for fine-grained classification.

### 3.4 Data splitting

Our dataset was partitioned into three different subsets to facilitate robust model development and evaluation, as follows:

- 70% for training, used to learn the model parameters.
- 10% for validation, used for hyperparameter tuning and for early-stopping training.
- 20% for testing, reserved exclusively for final performance evaluation.

The split was performed using a randomized sampling strategy to ensure that each subset maintained a representative distribution of the eight Rutab classes. This approach helps preserve class balance and ensures that all Rutab types are present across training, validation, and testing sets, thereby supporting generalizability and fair evaluation.

### 3.5 Model training

**Figure 3:**
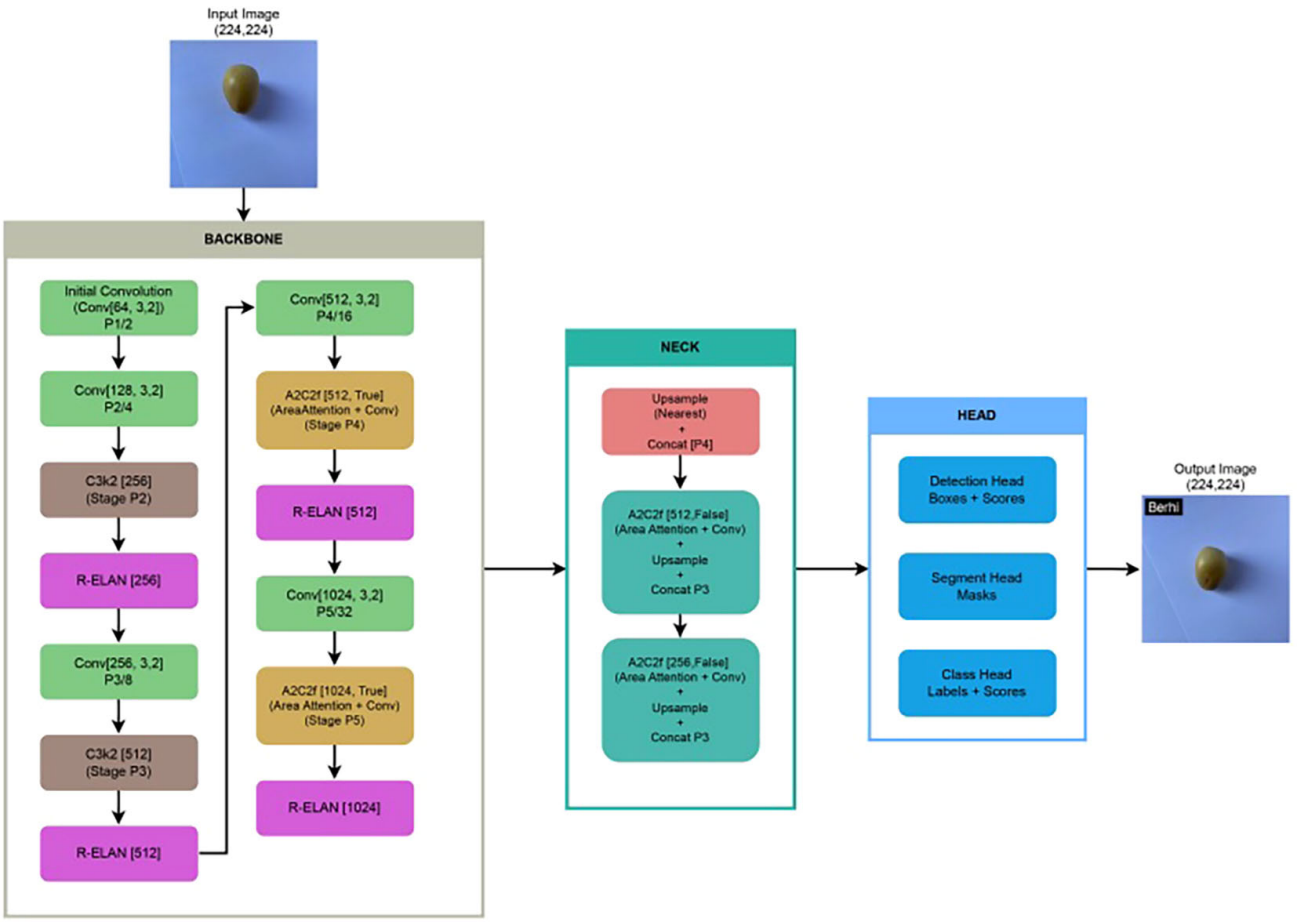
Architecture of our fine-tuned YOLOv12 model applied to our collected Rutab dataset, illustrating key components such as backbone, neck, and head, along with modifications introduced during fine-tuning for improved classification performance.

To develop a robust classification system for Rutab date varieties, we adopted and fine-tuned five well-established deep learning architectures. Each model was evaluated under the same experimental conditions to determine the most effective solution for deployment in the mobile application. The selected models are: Convolutional Neural Networks (CNNs) (LeCun et al., 1998), ResNet-50 (He et al., 2016), EfficientNet (Tan and Le, 2019), YOLOv8 (Varghese and Sambath, 2024), and YOLOv12 (Tian et al., 2025). A brief overview of each architecture is provided below:

- Convolutional Neural Networks (CNNs) (LeCun et al., 1998) are a category of deep learning architectures tailored for image analysis. They utilize convolutional layers to extract spatial hierarchies of features from input images, followed by pooling and fully connected layers for classification tasks. CNNs have achieved notable success in areas such as object recognition, facial identification, and handwritten digit classification.
- Residual Networks (ResNet-50) (He et al., 2016) introduce “skip connections” that allow the model to bypass certain layers, effectively addressing the vanishing gradient issue. This innovation enables the training of much deeper networks without compromising performance. ResNet-50, consisting of 50 layers, is widely adopted in image classification tasks and is frequently used as a feature extractor in computer vision applications.
- EfficientNet (Tan and Le, 2019) represents a class of CNN architectures that employ a compound scaling strategy to proportionally adjust the model’s depth, width, and resolution. This balanced scaling results in a highly efficient architecture that achieves strong accuracy while using fewer parameters and less computation than traditional models. Its lightweight nature makes it ideal for deployment in mobile and resource-constrained environments.
- You Only Look Once (YOLO) (Redmon et al., 2016; Varghese and Sambath, 2024; Tian et al., 2025) is a state-of-the-art real-time object detection framework. Unlike conventional approaches that apply classification within a sliding window or region proposal mechanism, YOLO reformulates object detection as a single end-to-end regression task. It simultaneously predicts bounding box coordinates and class probabilities in a single forward pass, resulting in high-speed inference and strong accuracy. For the adopted YOLOv12 model (Jegham et al., 2025; Tian et al., 2025), we utilized its classification variant. **Figure 3** illustrates the architecture of YOLOv12, highlighting the detailed structure of its backbone, neck, and head components.

Each of these models was fine-tuned on the collected Rutab dataset and evaluated using the same evaluation metrics, enabling a rigorous comparison of their effectiveness for our application.

### 3.6 Model evaluation

To evaluate and compare the performance of each fine-tuned state-of-the-art model, we employed three widely used classification metrics: precision, recall, and the F1-score. The results of this evaluation directly informed our selection of the optimal model for integration into the final mobile application. The evaluation metrics are described as follows:

Precision: Precision measures the proportion of correctly identified Rutab samples among all instances predicted as a particular class. The formula for precision is given by:


$$\text{Precision}=\frac{TP}{TP+FP}.$$

Recall: Recall assesses the proportion of actual Rutab samples that were correctly identified by the model. The formula for recall is:


$$\text{Recall}=\frac{TP}{TP+FN}.$$

F1-Score: The F1-score represents the harmonic mean of precision and recall, offering a unified measure that balances the trade-off between false positives and false negatives. It is calculated using the following formula:


$$\text{F}1-\text{Score}=2\times \frac{Precision\times Recall}{Precision+Recall}.$$

### 3.7 Hyperparameter settings

In this section, we explicitly present the key hyperparameters used for training the selected deep learning models. A batch size of 16 and an initial learning rate of 0.001 were consistently applied across all models. The number of training epochs varied by model, based on preliminary experiments and the point at which validation performance converged: 50 epochs for both the CNN and YOLOv8, 75 epochs for ResNet-50, and 100 epochs for EfficientNetB0 and YOLOv12. These choices reflect an empirical balance between training time and performance stability. **Table 3** provides a summary of the training hyperparameter configurations employed for all models evaluated in this study.

**Table 3:** A summary of the training hyperparameter settings used for all deep learning models evaluated in this study.

Parameter	CNN	ResNet-50	EfficientNetB0	YOLOv8	YOLOv12
Epochs	50	75	100	50	100
Batch Size	16	16	16	16	16
Learning Rate	0.001	0.001	0.001	0.001	0.001
Optimizer	Adam	Adam	Adam	SGD	Adam

### 3.8 Mobile application development

**Figure 4:**
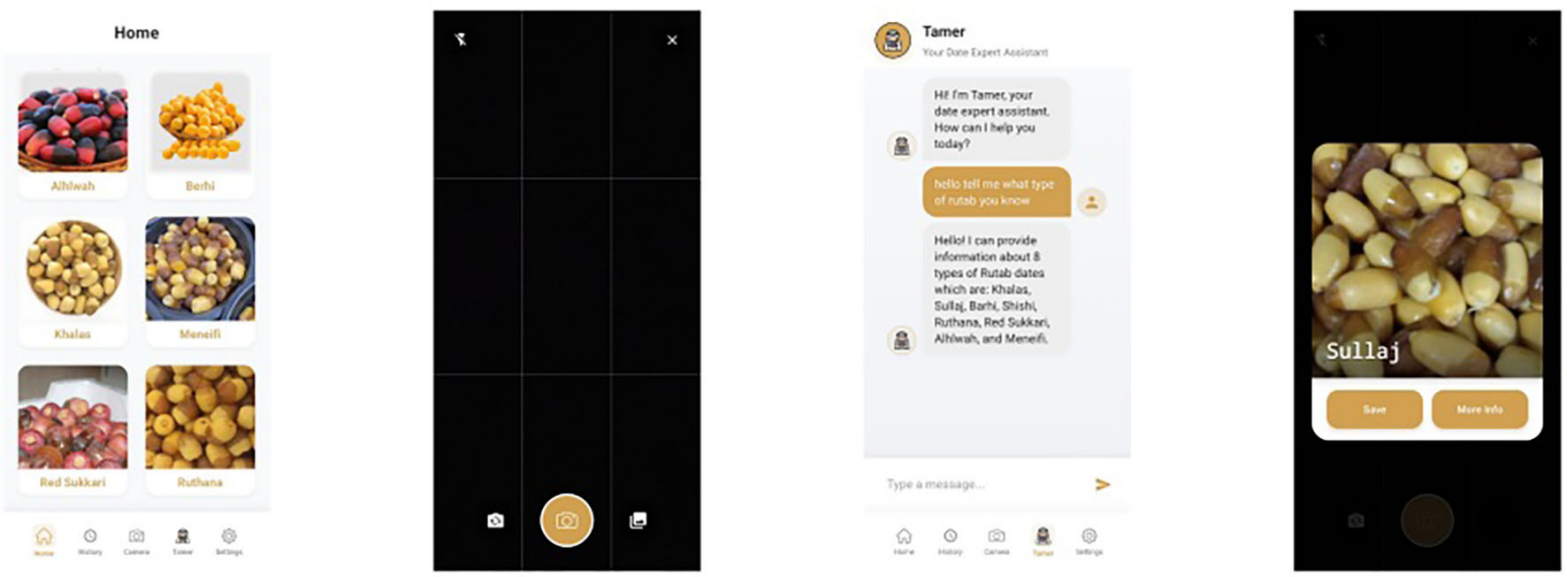
Sample screenshots from our developed mobile application, showcasing the integration of the best-performing classification model—YOLOv12 in our case—demonstrate the system’s real-time capability in accurately identifying Rutab date varieties.

Our objective extends beyond theoretical exploration; therefore, we have integrated the best-performing model —YOLOv12— into a fully developed, user-friendly mobile application. This application is publicly available, enabling users to classify Rutab date varieties in real-time, thereby bridging the gap between research and practical use. **Figure 4** presents sample screenshots of the deployed mobile application, demonstrating its real-time classification capability and intuitive interface.

### 3.9 Software environment

The data preprocessing stage was implemented in Python using the Pillow library, where all images were resized to a unified resolution of 224 × 224 pixels to standardize input dimensions for training. Data augmentation was also performed using Pillow.

Model training was conducted using both PyTorch and TensorFlow frameworks. PyTorch was used for ResNet, RNN, and YOLO implementations (with Ultralytics serving as the backend for YOLO). TensorFlow was used for training EfficientNet and CNN models. For mobile application deployment, the system was built using Flutter SDK with Dart, incorporating Hive for local storage, Camera and Image Picker packages for image input, an HTTP client for API communication, and OpenAI GPT-4 API integration for intelligent user interaction.

## 4 Results and analysis

This section details the experimental outcomes, beginning with a performance comparison of the evaluated models on the Rutab date classification task. We then analyze the confusion matrix of the top-performing model to assess its per-class accuracy and detail the key hyperparameter settings used to achieve these results.

### 4.1 Comparative performance analysis

**Table 4** presents a comparative evaluation of five deep learning models based on their performance on our test dataset. The baseline CNN model yielded the lowest scores across all metrics, reflecting its limited capacity for complex feature extraction. Both ResNet-50 and EfficientNetB0 demonstrated improved performance, with EfficientNetB0 slightly outperforming ResNet-50 in precision and recall, though both models achieved the same F1-score.

**Table 4:** A comparative analysis of the five deep learning architectures based on their performance on our test dataset.

Model	Precision	Recall	F1-score
CNN	44.5%	47.5%	44.8%
ResNet-50	69.9%	68.7%	68.5%
EfficientNetB0	70.6%	70%	68.5%
YOLOv8	84.7%	82.5%	82.4%
YOLOv12	93%	93%	93%

The results indicate that YOLOv12 outperforms competing models across all three evaluation metrics: Precision, Recall, and F-1 score.

**Figure 5:**
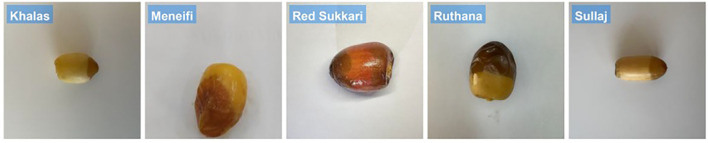
Sample classification outputs generated by the YOLOv12 model, illustrating the predicted Rutab varieties along with their corresponding class labels on the test images.

A substantial performance gain is observed with YOLOv8, which achieved over 82% in both precision and recall, highlighting its effectiveness in object detection tasks. Notably, YOLOv12 outperformed all other models, achieving 93% across precision, recall, and F1-score. This consistent and significant improvement underscores YOLOv12’s robustness and accuracy, justifying its inclusion in our developed mobile application. **Figure 5** presents sample classification results from the YOLOv12 model, showing the predicted Rutab varieties with their class labels on test images.

To evaluate model efficiency, we report both training and inference times across all architectures. Training durations varied significantly depending on model complexity and number of epochs: CNN required 25 seconds per epoch, EfficientNet took 24 seconds per epoch, ResNet-50 required 163 seconds per epoch, YOLOv8 finished in 19 seconds per epoch, and YOLOv12 trained for around 20 seconds per epoch. In terms of inference speed, YOLO models were the fastest (104 images/sec), followed by EfficientNet (37 images/sec), ResNet-50 (23 images/sec), and CNN (19 images/sec). These results highlight the trade-offs between model complexity, training time, and real-time performance, with YOLO models offering the most favorable balance for practical applications. To contextualize these metrics, model training was performed on a workstation equipped with an NVIDIA GeForce RTX 3080 GPU (10 GB VRAM), an Intel Core i7 CPU, and 32 GB RAM.

### 4.2 In-depth analysis: YOLOv12 performance

**Figure 6:**
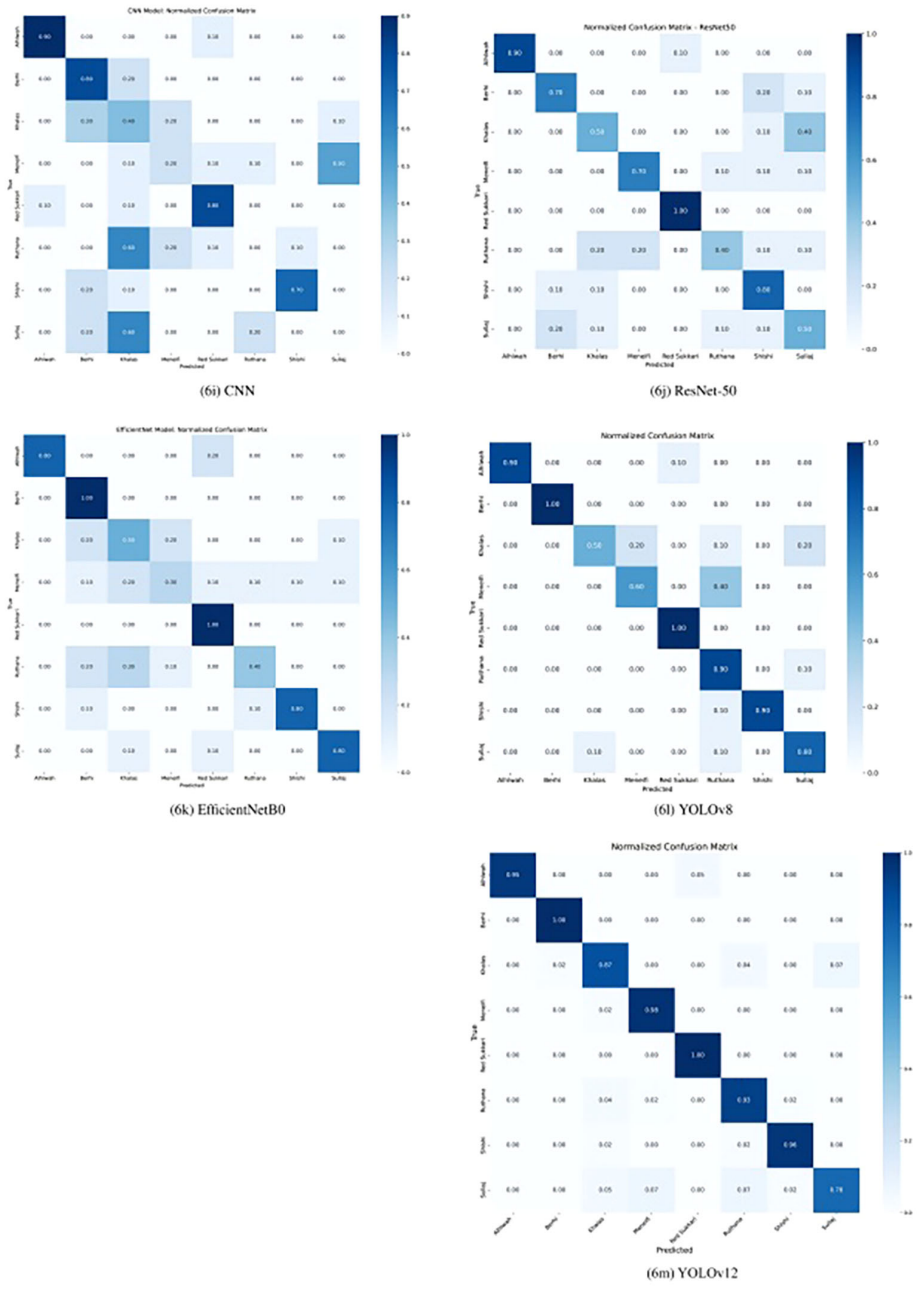
The confusion matrices for all evaluated models on the test dataset, highlighting YOLOv12 as the best-performing model.

Across the five evaluated models—CNN, EfficientNetB0, ResNet-50, YOLOv8, and YOLOv12—the confusion matrices reveal distinct patterns in classification performance for Rutab date varieties, as shown in **Figure 6**. YOLOv12 outperforms all previous models, achieving near-perfect recall across most classes: 100% for Berhi, and Red Sukkari; 98% for Menefi; 96% for Shishi; 95% for Alhlwah; and 93% for Ruthana. It also significantly improves classification of Khalas (87% recall) and Sullaj (78% recall), which were previously challenging for other models. YOLOv8 had already shown strong performance, especially on Berhi, Red Sukkari, Ruthana, Alhlwah, and Shishi, but YOLOv12 further reduces confusion and boosts accuracy across all classes. EfficientNetB0 and ResNet-50 showed moderate success, with EfficientNet performing well on Berhi and Red Sukkari, while ResNet handled Meneifi more effectively. The CNN model struggled significantly, failing to correctly classify Ruthana and Sullaj altogether and showing high misclassification rates among visually similar brown varieties like Khalas and Meneifi.

Common confusion patterns emerged across models, particularly among the yellow-colored varieties—Khalas, Sullaj, Meneifi, and Ruthana—due to their similar appearance at the Rutab stage. Alhlwah and Red Sukkari also showed minor confusion, likely because of their shared yellow-golden hue. YOLOv12 demonstrated the strongest ability to separate these classes, with minimal cross-class misclassification: for example, only 5% of Alhlwah were misclassified, and Sullaj confusion was reduced to small percentages across Khalas, Meneifi, Ruthana, and Shishi. Compared to YOLOv8, which still showed some confusion between Khalas and Sullaj, YOLOv12’s attention-centric architecture and refined feature aggregation clearly enhance its ability to distinguish visually similar agricultural products. Overall, YOLOv12 proves to be the most robust and accurate model for Rutab date classification in our study.

**Figure 7:**
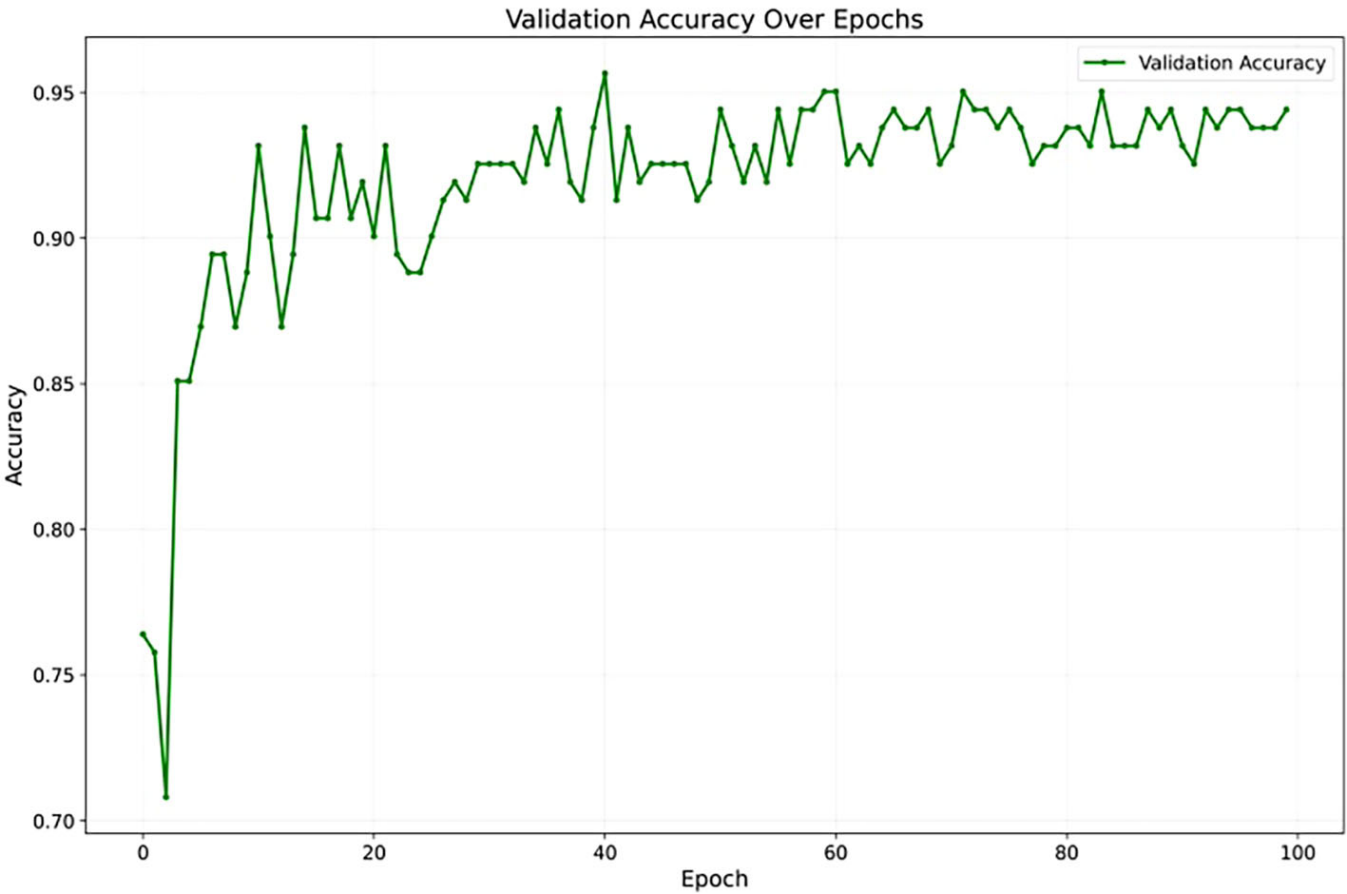
The validation accuracy over 100 epochs showing convergence of the YOLOv12 model.

**Figure 8:**
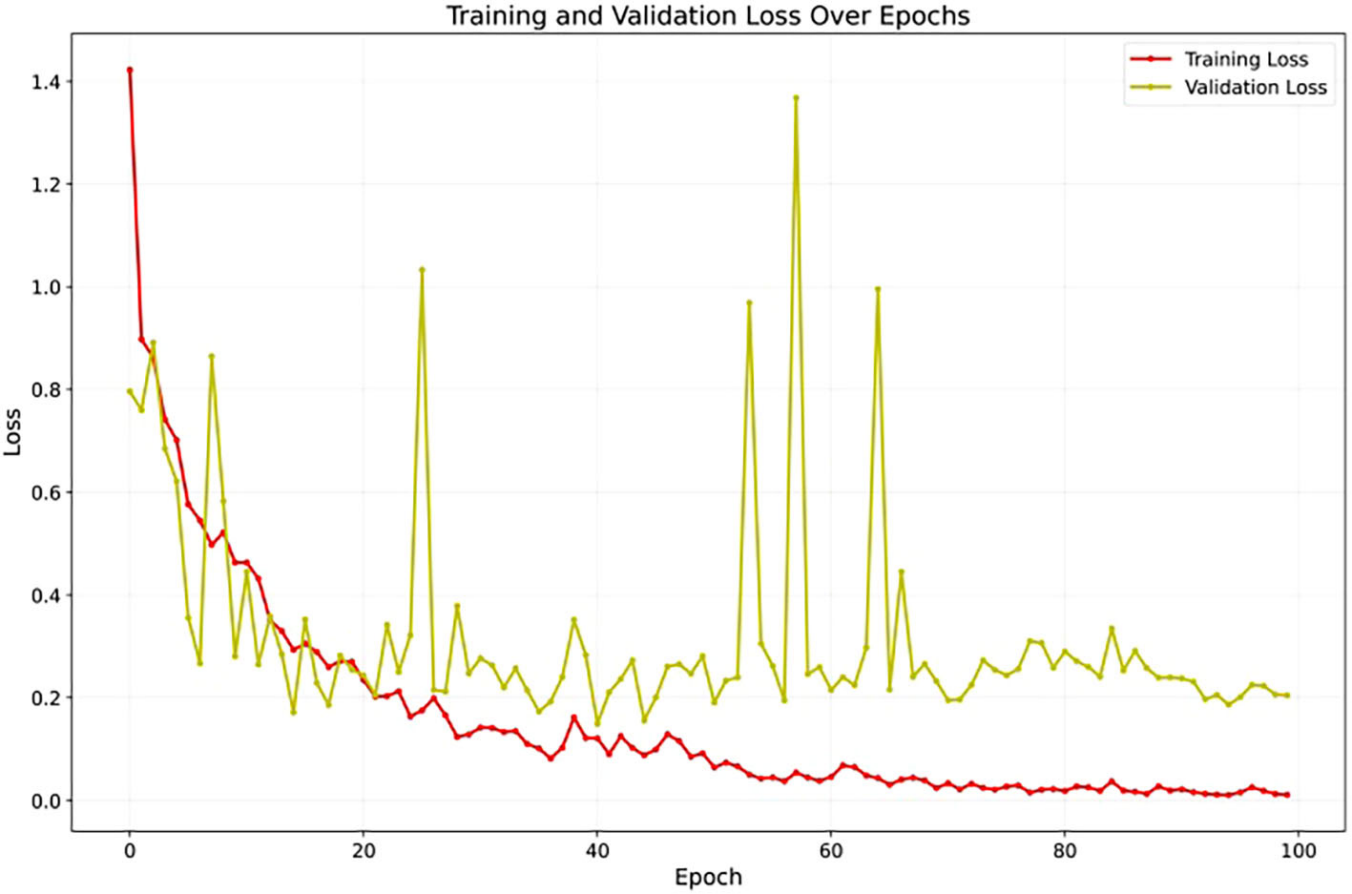
The training and validation loss over 100 epochs demonstrating stable model optimization.

To assess YOLOv12’s learning stability, **Figure 7** illustrates its accuracy on the validation set across training epochs. The curve shows a consistent upward trend, converging smoothly without unexpected fluctuations or divergence. The absence of instability and the sustained improvement in accuracy suggest that the model generalizes well to unseen data, offering strong evidence against overfitting throughout the training process. Also, **Figure 8** presents the training and validation loss curves over the same epochs. The loss steadily decreases for both sets, further confirming the model’s effective learning and stable training process without divergence.

Overall, the analysis confirms that YOLOv12 provides the best balance between accuracy and generalization. The model successfully learned to differentiate between all eight Rutab varieties — a challenging task given their high intra-class similarity and inter-class overlap, particularly among varieties with comparable color and shape profiles. Although YOLOv12 achieved strong performance on our Rutab dataset (93% precision, recall, and F1-score), the task remains inherently more difficult than Tamar classification. Prior studies on Tamar-stage classification often report higher accuracy because fully ripened fruits exhibit more distinctive visual features, such as darker hues and surface wrinkling, which aid model classification. These findings support the model’s deployment in our developed mobile application, where reliability and real-world performance are essential.

Despite the promising results achieved in this study, several limitations should be acknowledged. First, the dataset used—while carefully collected—remains relatively small, which may affect the generalizability of the model across broader contexts. Additionally, all images were collected under controlled indoor lighting conditions against a uniform white background, which do not fully reflect the variability encountered in real-world agricultural environments. Factors such as fluctuating natural light, diverse environmental conditions, and inconsistent backgrounds in farm scenarios could impact model performance.

## 5 Conclusions

This paper introduces a novel machine learning-based approach for the classification of “Rutab” dates, a ripening stage that holds both cultural and commercial importance yet remains largely underexplored in current literature. By focusing on this intermediate phase rather than the more commonly studied “Tamar” stage, our work provides new insights and tools for the classification of date varieties. The development of a custom image dataset and the successful implementation of deep learning models—particularly YOLOv12, which achieved a recall of 93%—demonstrate the potential of our pipeline in supporting agricultural and cultural applications. The integration of our classification pipeline into a mobile application further enhances its practical value, offering an accessible tool for farmers, researchers, and the public.

Although the proposed methodology demonstrates promising results in classifying Rutab date varieties, several avenues remain open for future exploration and enhancement. For example, the current dataset, while representative, is limited in size. Future work will focus on collecting a larger and more diverse dataset, encompassing various environmental conditions (e.g., lighting, background, occlusion) to improve the model’s robustness and generalization in real-world scenarios. In addition, the current classification framework includes eight commonly consumed Rutab types. Expanding the taxonomy to include lesser known or region-specific varieties will enhance the model’s comprehensiveness and cultural relevance.

Furthermore, future work will investigate the integration of explainable AI techniques to enhance transparency and trust in agricultural applications. Leveraging larger pre-trained models through transfer learning may further improve classification accuracy and generalization. Moreover, investigating multimodal approaches—such as combining image data with sensor inputs like moisture or temperature—could provide richer context and lead to more robust classification systems. These future directions aim to broaden the system’s applicability, improve performance, and contribute to the broader goal of using AI for agricultural and cultural heritage preservation.

## Data Availability

The raw data supporting the conclusions of this article will be made available by the authors, without undue reservation.
